# Security Analysis of a Passive Continuous-Variable Quantum Key Distribution by Considering Finite-Size Effect

**DOI:** 10.3390/e23121698

**Published:** 2021-12-19

**Authors:** Shengjie Xu, Yin Li, Yijun Wang, Yun Mao, Xiaodong Wu, Ying Guo

**Affiliations:** 1 School of Automation, Central South University, Changsha 410083, China; 206166@csu.edu.cn (S.X.); liyin@csu.edu.cn (Y.L.); xxywyj@csu.edu.cn (Y.W.); 2 School of Economics and Mangement, Beihua University, Jilin 132013, China

**Keywords:** passive, continuous-variable quantum key distribution, finite-size effect

## Abstract

We perform security analysis of a passive continuous-variable quantum key distribution (CV-QKD) protocol by considering the finite-size effect. In the passive CV-QKD scheme, Alice utilizes thermal sources to passively make preparation of quantum state without Gaussian modulations. With this technique, the quantum states can be prepared precisely to match the high transmission rate. Here, both asymptotic regime and finite-size regime are considered to make a comparison. In the finite-size scenario, we illustrate the passive CV-QKD protocol against collective attacks. Simulation results show that the performance of passive CV-QKD protocol in the finite-size case is more pessimistic than that achieved in the asymptotic case, which indicates that the finite-size effect has a great influence on the performance of the single-mode passive CV-QKD protocol. However, we can still obtain a reasonable performance in the finite-size regime by enhancing the average photon number of the thermal state.

## 1. Introduction

Quantum key distribution (QKD) solves the problem of sharing secure keys between two distant authenticated users (Alice and Bob). These two users can perform secure communications when such keys are established [1,2,3,4]. QKD has been divided into two main categories: one is discrete-variable (DV)QKD protocols [5,6], and the other is continuous-variable (CV) QKD schemes [7,8,9,10]. CV-QKD takes advantage of the quadrature components of the optical field to perform the key information distribution. Compared with DV-QKD, CV-QKD has better compatibility with existing optical communication systems and employs lower-cost light sources and detectors.

The Gaussian-modulated CV-QKD protocol making use of coherent states has attracted much attention because of its theoretical security [11,12,13,14,15,16,17,18] and its practicality [19,20,21,22]. In this protocol, the quantum state is traditionally prepared in an active manner: Gaussian distributed random numbers are firstly generated by Alice with the help of a true random number generator, then Alice can perform preparation of a coherent state and transmit it to Bob. The modulation method used in the Gaussian-modulated CV-QKD protocol is that Alice modulates the output of a laser by taking advantage of high-speed amplitude and phase modulators with requisite high extinction ratio. Because the modulation format of the Gaussian-modulated CVQKD scheme is relatively complex and the tolerable modulation error is small, it is necessary to make use of high extinction ratio modulators with good stability in this protocol [23].

However, the use of high extinction ratio modulators may present evident enhancement in cost, especially, creating an important challenge in the chip-integration in view of cost-effective silicon photonics technology [24]. The authors of [25] demonstrated on-chip modulators with high extinction ratio over 65 dB. The high-speed on-chip modulators needed in active QKD encoding schemes bring about significant cost, manufacturing time, and complexity. Consequently, it is important to study the potential of removing the modulators, which, when taken advantage of for encoding, may yield obvious reductions in cost and manufacturing time.

Recently, a passive-state preparation scheme in view of single-mode thermal source rather than high extinction ratio modulators has been proposed, whose aim is to simplify the implementation of CV-QKD [26]. By assuming that Alice’s QKD transmitter is trusted, it can take advantage of the well-established security proofs directly for Gaussian-modulated CV-QKD into passive CV-QKD protocol. This passive-state preparation scheme has been applied in CV quantum secret sharing [27] and measurement-device-independent CV-QKD [28,29]. More recently, an experimental study of the passive-state preparation protocol [30] and the local-oscillator-based passive CV-QKD scheme [31] have been proposed, which demonstrate the feasibility of passive CV-QKD in practical implementation. The practical implementations of a thermal source can be realized by employing a broadband-amplified spontaneous emission (ASE) source, which contains many spectral–temporal modes of independent thermal states [30,31]. Compared to a direct Gaussian modulation CV-QKD protocol, the passive CV-QKD scheme with practical implementations of a thermal source has its own advantages, namely, this protocol waives the necessity of utilizing high-extinction ratio amplitude and phase modulators, which may yield significant reductions in cost. The interesting extension of this work may be found in quantum algorithms [32,33], quantum computational speed [34], and quantum communication networks [35]. The security analysis of single-mode passive CV-QKD in asymptotic scenarios has been presented [26]. Nevertheless, the utility of single-mode passive CV-QKD protocol in finite-size regimes has never been analyzed.

In this paper, we perform security analysis of single-mode passive CV-QKD protocol by considering finite-size effect. Here, only the reverse reconciliation scheme is taken into consideration, since the direct reconciliation scheme can be analyzed in a similar way. The numerical simulations of the scheme are conducted by employing block lengths between $10^{7}$ and $10^{11}$. When the amount of data samples taken advantage of to perform parameter estimation is large, the performance of single-mode passive CV-QKD protocol in a finite-size regime will approach that in the asymptotic scenario.

The paper is structured as follows. In Section 2, we introduce the main idea of the single-mode passive CV-QKD protocol. In Section 3, we perform the security analysis with numerical simulations by considering an asymptotic case and the finite-size effect. Finally, conclusions are drawn in Section 4.

## 2. Passive CV-QKD Protocol

**Figure 1:**
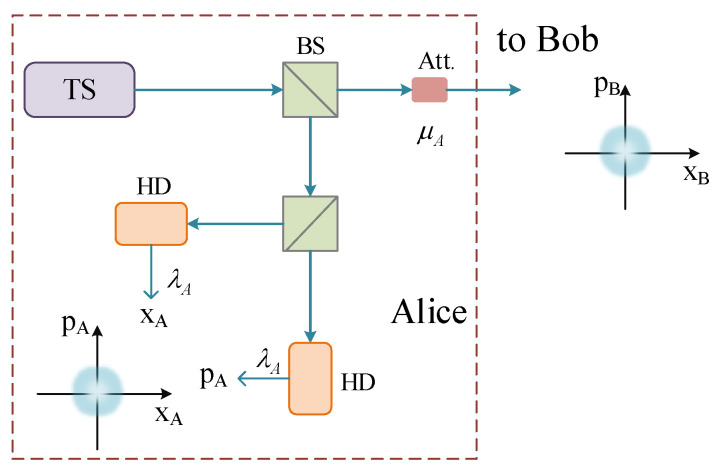
Single-mode passive CV-QKD protocol [26]. HD, homodyne detector; BS, beam splitter; Att., optical attenuator. Here, we employ a beam splitter with a transmittance of $\omega_{M}$ to model the efficiency of the homodyne detector.

The setup of the passive CV-QKD is shown in Figure 1. This scheme makes use of the intrinsic field fluctuations of a thermal source to generate a secure quantum key [26]. As illustrated in Figure 1, Alice makes use of a balanced beam splitter to split the output of a thermal source into two spatial modes. One mode is locally measured by Alice with the help of conjugate homodyne detection, then the other mode is transmitted to Bob through an optical attenuator. In order to make an estimate of the quadrature values of the outgoing mode, it is necessary to achieve the Gaussian-distributed random numbers $\left(x_{A},p_{A}\right)$. Therefore, the local measurement owned by Alice is scaled down numerically via a factor of $\lambda_{A}$, which can obtain Alice’s desired modulation variance value $V_{A}$ with a proper combination of source intensity and optical attenuation. Besides, it has been proved that the passive CV-QKD protocol is equivalent to the GMCS QKD protocol in terms of security [26].

It is noteworthy that the excess noise caused by the quantum state preparation has an important effect on the performance of the passive CV-QKD protocol; the mutual information between Alice and Bob is associated with Alice’s uncertainties on the quadrature of the outgoing mode. According to the uncertainty principle in quantum mechanics, the minimum uncertainty on either quadrature value of the outgoing mode (equal to 1) can be achieved by Alice. In the passive CV-QKD protocol (illustrated in Figure 1), Alice’s uncertainty on the outgoing mode is given by [26]

(1) $$\Lambda_{A}=\frac{2\mu_{A}}{\omega_{M}}\left(1-\frac{\omega_{M}}{2}+\nu_{el}\right)+1,$$

where $\mu_{A}$ represents the transmittance of the optical attenuator, and $\omega_{M}$ and $\nu_{el}$ stand for the efficiency and noise variance of detector owned by Alice, respectively. According to Equation (1), the excess noise caused by the passive state preparation can be calculated as

(2) $$\xi_{A}=\Lambda_{A}-1=\frac{2\mu_{A}}{\omega_{M}}\left(1-\frac{\omega_{M}}{2}+\nu_{el}\right).$$

Making use of the relation $V_{A}=\mu_{A}m_{0}$, Equation (2) is revised as

(3) $$\xi_{A}=\frac{2V_{A}}{\omega_{M}m_{0}}\left(1-\frac{\omega_{M}}{2}+\nu_{el}\right),$$

where $m_{0}$ represents an average photon number of a thermal source owned by Alice.

**Figure 2:**
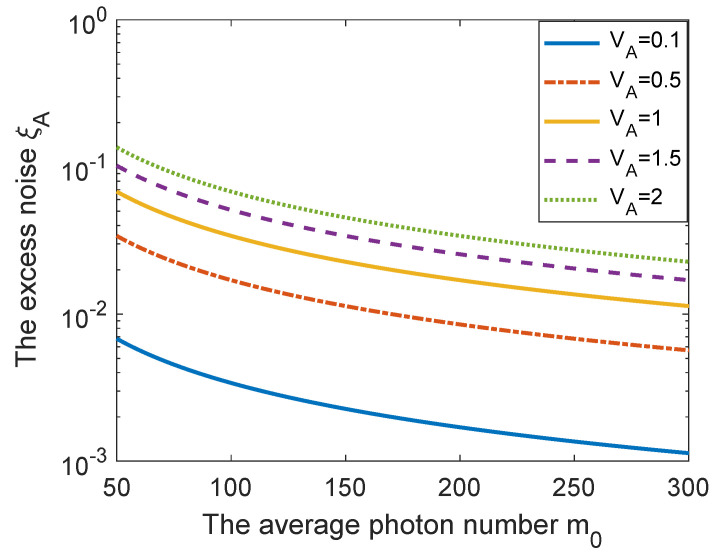
The excess noise $\xi_{A}$ as a function of the average photon number $m_{0}$ with different modulation variance values $V_{A}$. Simulation parameters are $\omega_{M}=0.5$, $\nu_{el}=0.1$ [26].

Considering the fact that the excess noise due to the passive state preparation $\xi_{A}$ always exists in the single-mode passive CV-QKD protocol, it is necessary to analyze the excess noise $\xi_{A}$ to achieve desired performance. The excess noise $\xi_{A}$ as a function of the average photon number $m_{0}$ with different modulation variance values $V_{A}$ is shown in Figure 2. One can find that the larger the average photon number $m_{0}$ is, the smaller the excess noise $\xi_{A}$ is, especially for the low value of modulation variance. Besides, the reduction of modulation variance $V_{A}$ can also effectively restrain the excess noise $\xi_{A}$. According to [26], a typical value of $V_{A}$ = 1 can be satisfied by a practical broadband thermal source. Therefore, the modulation variance value of $V_{A}$ used in the following simulations is set to $V_{A}$ = 1.

It is necessary to point out that [30,31] employ broadband-amplified spontaneous emission source, which contains many spectral–temporal modes of independent thermal states, and is different from the single-mode thermal source. In [30,31], the excess noise caused by the passive state preparation using multimode thermal source is related with the mode-overlap coefficient. However, it is not necessary to consider the relationship between the excess noise caused by the passive state preparation and the mode-overlap coefficient with the use of single-mode thermal source shown in our protocol.

## 3. Security Analysis

In this section, we perform security analysis of passive CV-QKD protocol by taking both asymptotic case [15] and finite-size regime [16] into consideration.

### 3.1. Asymptotic Security of Passive CV-QKD Protocol

Here, we calculate the asymptotic secure key rate of the passive CV-QKD protocol with reverse reconciliation, which is given by [13,36]

(4) $$K_{asy}=\beta I\left(A:B\right)-\chi \left(E\right),$$

where β represents the reconciliation efficiency, I(A:B) represents the Shannon mutual information between Alice and Bob, and χ(E) represents the Holevo bound of the information owned by Eve. Here, channel losses is assumed as α=0.2 dB/km. The transmittance is given by

(5) $$T=10^{\frac{-\alpha L}{10}},$$

where *L* represents the fiber length in kilometers.

We now calculate the noise added by Bob’s detector for conjugate homodyne detection, which is expressed as [36]

(6) $$\chi_{het}=\left[1+\left(1-\omega_{M}\right)+2\nu_{el}\right]/\omega_{M},$$

where we have made an assumption that the performance of Bob’s detector is the same as that of Alice’s.

For the channel-added noise referred to the channel input, it can be calculated as

(7) $$\chi_{line}=\frac{1}{T}-1+\xi_{A}+\xi_{0},$$

where $\xi_{A}$ stands for the excess noise caused by Alice’s passive state preparation (shown in Equation (3)). $\xi_{0}$ stands for other sources of untrusted noise.

Based on the above analysis, we can present the overall noise referred to the channel input, which is given by

(8) $$\chi_{tot}=\chi_{line}+\frac{\chi_{het}}{T}.$$

Considering that both quadratures can be taken advantage of to make the generation of the secure key, we can thus determine the mutual information between Alice and Bob, which is given by

(9) $$I\left(A:B\right)=log_{2}\frac{V+\chi_{tot}}{1+\chi_{tot}},$$

where $V=V_{A}+1$.

Since we adopt a reverse reconciliation scheme to calculate the secret key rate of the passive CV-QKD protocol, the parameter χ(E)=χ(B:E). Here, χ(B:E) stands for the Holevo bound between Eve and Bob. In order to make an estimation of parameter χ(B:E), the realistic noise mode shown in [10] was adopted, which has been utilized widely in CV-QKD experiments [10,13,19,37,38]. Based on this model, we can calculate the parameter χ(B:E) as

(10) $$\chi \left(B:E\right)=\sum_{i=1}^{2}G\left(\frac{\rho_{i}-1}{2}\right)-\sum_{i=3}^{5}G\left(\frac{\rho_{i}-1}{2}\right),$$

where $G\left(x\right)=\left(x+1\right)\log_{2}\left(x+1\right)-x\log_{2}x$.

(11) $$\rho_{1,2}^{2}=\frac{1}{2}\left(\Delta \pm \sqrt{\Delta^{2}-4D}\right),$$

where

(12) $$\begin{array}{cc} \Delta & =V^{2}\left(1-2T\right)+2T+T^{2}{\left(V+\chi_{line}\right)}^{2}, \end{array}$$

(13) $$\begin{array}{cc} D & =T^{2}{\left(V\chi_{line}+1\right)}^{2}. \end{array}$$

(14) $$\rho_{3,4}^{2}=\frac{1}{2}\left(A\pm \sqrt{A^{2}-4B}\right),$$

where

(15) $$\begin{array}{cc} A & =\frac{1}{{\left[T\left(V+\chi_{tot}\right)\right]}^{2}}\left\{\Delta \chi_{het}^{2}+D+1+2\chi_{het}\left[V\sqrt{D}+T\left(V+\chi_{line}\right)\right]+2T\left(V^{2}-1\right)\right\}, \end{array}$$

(16) $$\begin{array}{cc} B & ={\left[\frac{V+\sqrt{D}\chi_{het}}{T(V+\chi_{tot})}\right]}^{2}, \end{array}$$

(17) $$\rho_{5}=1.$$

**Figure 3:**
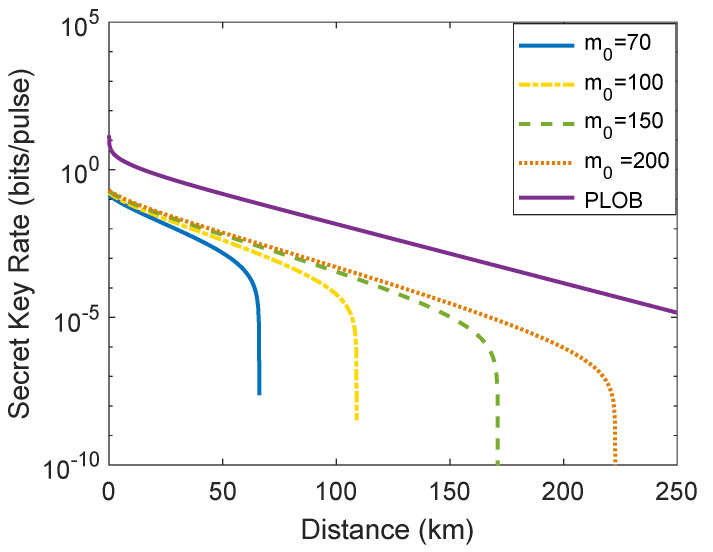
The relationship between the asymptotic secret key rate and the distance under four different average photon numbers: $m_{0}$ = 70, $m_{0}$ = 100, $m_{0}$ = 150, and $m_{0}$ = 200. Simulation parameters are $V_{A}=1$, $\xi_{0}=0.01$, $\omega_{M}=0.5$, $\nu_{el}=0.1$, and reconciliation efficiency β=0.95 [26].

In the following, we illustrate the relationship between the asymptotic secret key rate and the transmission distance under four different average photon numbers $m_{0}$ = 70, $m_{0}$ = 100, $m_{0}$ = 150, and $m_{0}$ = 200. The Pirandola–Laurenza–Ottaviani–Banchi (PLOB) bound is also plotted in Figure 3, which illustrates the ultimate limit of repeaterless quantum communication [39]. From Figure 3, we can observe that the performance of the passive CV-QKD protocol in terms of asymptotic secret key rate and transmission distance is enhanced by increasing the average output photon number $m_{0}$. As a matter of course, the performance of the single-mode passive CV-QKD protocol becomes more and more close to the PLOB bound with the increase of $m_{0}$. We can find the reason from Equation (3), namely, with a desired $V_{A}$, the larger average output photon number $m_{0}$, the smaller the excess noise $\xi_{A}$ introduced by Alice. In addition, one can find that the maximum transmission distance is over 100 km when $m_{0}$ = 100. That is to say, we can perform efficient implementation of the passive CV-QKD protocol with $m_{0}$ above 100.

**Figure 4:**
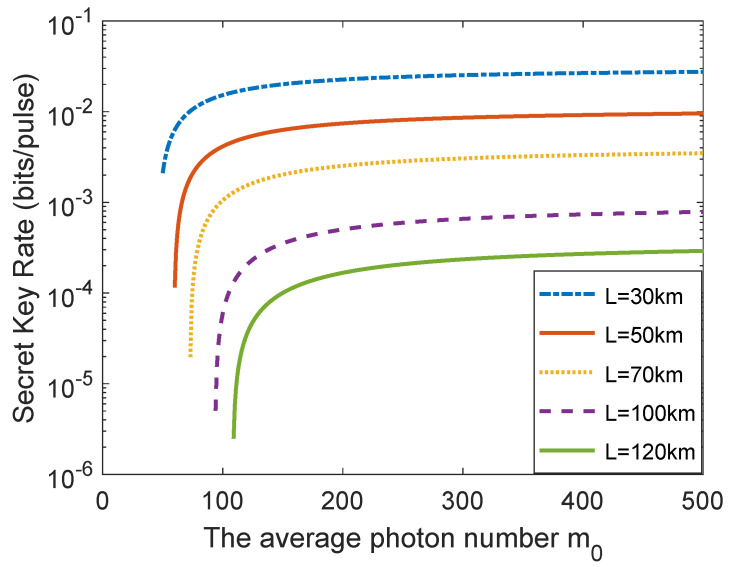
The asymptotic secret key rate as a function of average photon number $m_{0}$ under different distances. Simulation parameters are $V_{A}=1$, $\xi_{0}=0.01$, $\omega_{M}=0.5$, $\nu_{el}=0.1$, reconciliation efficiency β=0.95 [26].

Figure 4 illustrates the asymptotic secret key rate as a function of average photon number $m_{0}$ under different distances. From Figure 4, one can observe that the asymptotic secret key rate of the single-mode passive CV-QKD protocol grows fast in the interval [60,200]; nevertheless, it enhances slowly in the interval [200,500]. This indicates that when the average photon number $m_{0}$ reaches a certain value, the performance improvement is inapparent with continuing increase of average photon number $m_{0}$.

### 3.2. Security of Passive CV-QKD in Finite-Size Scenario

In the above analysis, we show the calculation of the asymptotic secret key rate of the passive CV-QKD protocol based on an assumption that Alice and Bob can take advantage of infinitely many signals to make the exchange. Nevertheless, it is impossible to achieve in practice, as the length of the practical secure key is limited. Consequently, it is necessary to perform security analysis of the passive CV-QKD scheme by considering the finite-size effect. The finite-size secret key rate of the single-mode passive CV-QKD protocol with reverse reconciliation is given by [16]

(18) $$K_{fini}=\frac{f}{F}\left[\beta I\left(A:B\right)-\chi_{\epsilon_{PE}}\left(B:E\right)-\Delta \left(f\right)\right],$$

where the meanings of β and I(A:B) are shown above. *F* represents the total exchanged signals and *f* is the number of signals which are used to generate secure key, and the leftover signal P=F−f is taken advantage of to perform parameter estimation. $\epsilon_{PE}$ stands for the failure probability of parameter estimation, and Δ(f) is associated with the security of the privacy amplification, which is given by

(19) $$\Delta \left(f\right)=\left(2dim\Psi_{B}+3\right)\sqrt{\frac{\log_{2}\left(2/\bar{\epsilon }\right)}{f}}+\frac{2}{f}\log_{2}\left(1/\epsilon_{PB}\right),$$

where $\bar{\epsilon }$ is assumed to be the smoothing parameter, $\epsilon_{PB}$ represents the failure probability that exists in the privacy amplification procedure, and $\Psi_{B}$ stands for the Hilbert space corresponding to the raw key owned by Bob. Here, $dim\Psi_{B}=2$ because the raw key is encoded on binary bits.

In order to perform the security analysis of the single-mode passive CV-QKD protocol in a finite-size regime, it is important to make calculation of $\chi_{\epsilon_{PE}}\left(B:E\right)$ by employing a covariance matrix assumed as $\Upsilon_{\epsilon_{PE}}$, which makes the secret key rate of the single-mode passive CV-QKD protocol minimum exist under a probability of $1-\epsilon_{PE}$. Through using *P* couples of correlated variables ${\left(x_{i},y_{i}\right)}_{i=1,2,\cdot \cdot \cdot ,P}$, we can achieve the covariance matrix $\Upsilon_{\epsilon_{PE}}$. To perform analysis of these correlated variables, we adopt a normal model, which is shown as follows:

(20) y=tx+z,

where $t=\sqrt{T}$ and *z* follow a centered normal distribution with variance $\vartheta^{2}=1+T\left(\xi_{A}+\xi_{0}\right)$. According to Equation (20), the data owned by Alice and Bob can be connected. The covariance matrix $\Upsilon_{\epsilon_{PE}}$ is given by

(21) $$\Upsilon_{\epsilon_{PE}}=\left(\begin{array}{cc} \left(V_{A}+1\right)I_{2} & t_{min}Z\sigma_{z}\\ t_{min}Z\sigma_{z} & \left(t_{min}^{2}V_{A}+\vartheta_{max}^{2}\right)I_{2} \end{array}\right),$$

where $t_{min}$ and $\vartheta_{max}^{2}$ stand for the minimum of *t* and maximum of $\vartheta^{2}$ compatible with sampled couples, except with probability $\epsilon_{PE}/2$, and $Z=\sqrt{V_{A}^{2}+2V_{A}}$. After that, we can obtain the maximum-likelihood estimators $\hat{t}$ and ${\hat{\vartheta }}^{2}$, which are calculated as

(22) $$\hat{t}=\frac{\sum_{i=1}^{P}x_{i}y_{i}}{\sum_{i=1}^{P}x_{i}^{2}}\;and\;{\hat{\vartheta }}^{2}=\frac{1}{P}\sum_{i=1}^{P}{\left(y_{i}-\hat{t}x_{i}\right)}^{2}.$$

Distributions followed by the maximum-likelihood estimators $\hat{t}$ and ${\hat{\vartheta }}^{2}$ are, respectively, given by

(23) $$\hat{t}∼N\left(t,\frac{\vartheta^{2}}{\sum_{i=1}^{P}x_{i}^{2}}\right)\;and\;\frac{P{\hat{\vartheta }}^{2}}{\vartheta^{2}}∼\chi^{2}\left(P-1\right),$$

which indicate that $\hat{t}$ and ${\hat{\vartheta }}^{2}$ are independent for each other. In view of [16], we respectively show the expressions of $t_{min}$ and $\vartheta_{max}^{2}$, which are given by

(24) $$\begin{array}{cc} t_{min} & \approx \hat{t}-z_{\epsilon_{PE}/2}\sqrt{\frac{{\hat{\vartheta }}^{2}}{PV_{A}}},\\ \vartheta_{max}^{2} & \approx {\hat{\vartheta }}^{2}+z_{\epsilon_{PE}/2}\frac{\sqrt{2}{\hat{\vartheta }}^{2}}{\sqrt{P}}, \end{array}$$

where $z_{\epsilon_{PE}/2}$ is such that $1-\mathrm{erf}\left(z_{\epsilon_{PE}/2}/\sqrt{2}\right)/2=\epsilon_{PE}/2$, and erf represents the error function defined as $\mathrm{erf}\left(x\right)=\frac{2}{\sqrt{\pi }}\int_{0}^{x}e^{-t^{2}}dt$. Making use of the expected values of $\hat{t}$ and ${\hat{\vartheta }}^{2}$, which are $E\left[\hat{t}\right]=\sqrt{T}$ and $E\left[{\hat{\vartheta }}^{2}\right]=1+T\left(\xi_{A}+\xi_{0}\right)$, one can perform calculation of $t_{min}$ and $\vartheta_{max}^{2}$ as

(25) $$\begin{array}{cc} t_{min} & \approx \sqrt{T}-z_{\epsilon_{PE}/2}\sqrt{\frac{1+T(\xi_{A}+\xi_{0})}{PV_{A}}},\\ \vartheta_{max}^{2} & \approx 1+T\left(\xi_{A}+\xi_{0}\right)+z_{\epsilon_{PE}/2}\frac{\sqrt{2}\left[1+T\left(\xi_{A}+\xi_{0}\right)\right]}{\sqrt{P}}. \end{array}$$

It is noteworthy that the error probabilities shown above are set to [16]

(26) $$\bar{\epsilon }=\epsilon_{PE}=\epsilon_{PB}=10^{-10}.$$

Making use of the derived bound $t_{min}$ and $\vartheta_{max}^{2}$, the finite-size secret key rate of single-mode passive CV-QKD protocol can be calculated.

**Figure 5:**
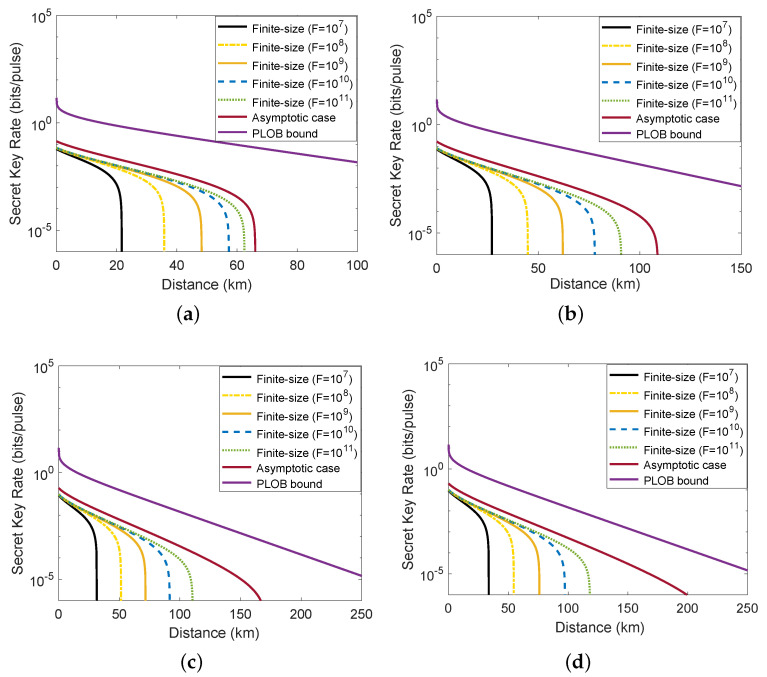
The relationship between the finite-size secret key rate of single-mode passive CV-QKD protocol and the distance: (**a**) $m_{0}$ = 70; (**b**) $m_{0}$ = 100; (**c**) $m_{0}$ = 150; (**d**) $m_{0}$ = 200. From left to right, the lines correspond to block lengths of $F=10^{7}$, $10^{8}$, $10^{9}$, $10^{10}$, and $10^{11}$. Other parameters are set to be the same as Figure 3.

Figure 5 illustrates the relationship between the finite-size secret key rate and the transmission distance under four different average photon numbers $m_{0}$. It is worth mentioning that the average output photon number $m_{0}$ we set in Figure 5a–d are 70, 100, 150, and 200. From left to right, the lines shown in Figure 5 correspond to block lengths of $10^{7}$, $10^{8}$, $10^{9}$, $10^{10}$, and the asymptotic case. Here, we plot the PLOB bound in all four subgraphs to make a detailed comparison. As shown in Figure 5, one can observe that the performance of the single-mode passive CV-QKD protocol in the finite-size regime is more pessimistic than that obtained in the asymptotic limit. This is in line with our expectations because a part of the exchanged signals needs to be made use of to perform parameter estimation instead of generating the secure key in the finite-size regime. Nevertheless, the performance of passive CV-QKD protocol in terms of secret key rate and maximum transmission distance in the finite-size regime becomes more and more close to that in the asymptotic case and the PLOB bound with the increase of the number of total exchanged signals. In addition, we can still achieve a reasonable performance in the finite-size scenario by improving the average photon number of the thermal state.

**Figure 6:**
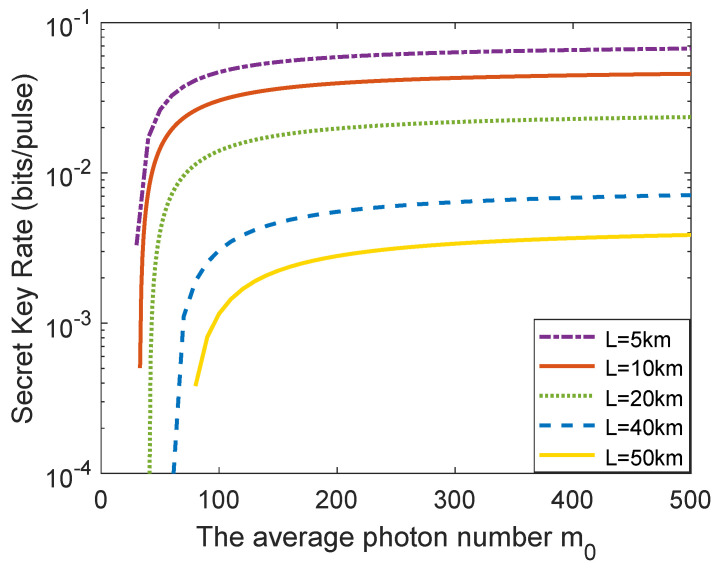
The relationship between the finite-size secret key rate and the average photon number $m_{0}$ with different distances. The block size is set to $F=10^{9}$. Other parameters are set to be the same as Figure 4.

The relationship between the finite-size secret key rate and the average photon number $m_{0}$ with different distances is shown in Figure 6. Here, the block size $F=10^{9}$ is used as an example to perform analysis since other block size cases can be analyzed in the same way. It can be seen that the finite-size secret key rate of the single-mode passive CV-QKD protocol grows fast in the interval [30,200]; however, it increases slowly in the interval [200,500]. The results make it clear that when the average photon number $m_{0}$ reaches a certain value, the finite-size secret key rate enhancement is inapparent with continuing increase of the average photon number $m_{0}$.

**Figure 7:**
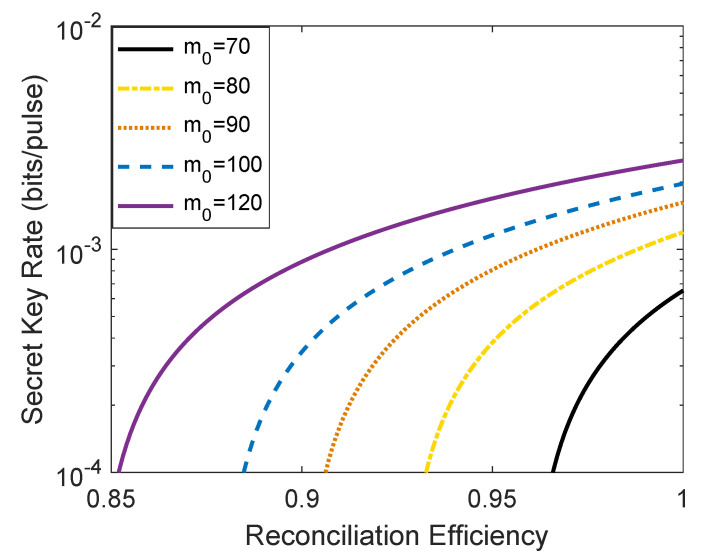
The relationship between the finite-size secret key rate of single-mode passive CV-QKD protocol and the reconciliation efficiency β. The block size is set to $F=10^{9}$. Other parameters are set to be the same as Figure 3.

The plot of Figure 7 shows the relations of the finite-size secret key rate and the reconciliation efficiency. Similar to Figure 6, here, we take the block size $F=10^{9}$ as an example to perform analysis since other block size cases can be analyzed in the same way. It can be seen that the usable range of the reconciliation efficiency β of the passive CV-QKD protocol in the finite-size regime expands with the enhancement of the average photon number $m_{0}$. For example, when $m_{0}$ = 70, the usable range of β is [0.96,1]. However, when $m_{0}$ = 100, the usable range of β is [0.88,1].

## 4. Conclusions

We performed the security analysis of single-mode passive CV-QKD protocol by considering the finite-size effect under collective attack. By taking advantage of the single-mode passive CV-QKD protocol in the finite-size regime of the secret key rate formula for numerical simulation, one can find the secret key rate and maximum transmission distance under the influence of the finite-size effect. Therefore, the performance of the single-mode passive CV-QKD protocol in a finite-size regime is more pessimistic than those achieved in asymptotic case. However, with the enhancement of the number of total exchanged signals, the secret key rate and maximum transmission distance in the finite-size regime becomes more and more close to those in asymptotic case and the PLOB bound. Our work focuses on the influence of the finite-size effect on the single-mode passive CV-QKD protocol, which shows more practical results than those achieved in asymptotic case.

## Data Availability

Data sharing not Applicable.
